# Intussusception in children aged under two years in India: Retrospective surveillance at nineteen tertiary care hospitals

**DOI:** 10.1016/j.vaccine.2020.04.059

**Published:** 2020-10-07

**Authors:** Manoja Kumar Das, Narendra Kumar Arora, Bini Gupta, Apoorva Sharan, K. Kameswari, P. Padmalatha, G. Rajendra Prasad, Jimmy Shad, J. Shyamala, S. Harish Kumar, Yoga Nagender, K. Sharmila, Rashmi Shad, Saurabh Garge, Lalit Bharadia, Atul Gupta, Jayanta K. Goswami, Kaushik Lahiri, Lalit Sankhe, Sushant Mane, Yogini Prasad Patwari, M.K. Ajayakumar, A. Santhosh Kumar, Rachita Sarangi, Bikasha Bihary Tripathy, S.S.G. Mohapatra, Subrat Kumar Sahoo, Vijayendra Kumar, Rakesh Kumar, Suman Sarkar, Ruchirendu Sarkar, Nihar Ranjan Sarkar, Ashish Wakhlu, Simmi K. Ratan, Anand P. Dubey, Neelam Mohan, Meera Luthra, Bhadresh R. Vyas, Harsh Trivedi, John Mathai, Cenita J. Sam, K. Jothilakshmi, Pavai Arunachalam, Javeed Iqbal Bhat, Gowhar Mufti, Bashir Ahmad Charoo, Pradeep K. Jena, Sanjib K. Debbarma, Sunil K. Ghosh, Mahesh K. Aggarwal, Pradeep Haldar, Patrick L.F. Zuber, Christine Maure, Jan Bonhoeffer, Arindam Ray

**Affiliations:** a The INCLEN Trust International, New Delhi, India; b Andhra Medical College, Vishakhapatnam, Andhra Pradesh, India; c Apollo Hospitals, Chennai, Tamil Nadu, India; d Apollo Hospital, Hyderabad, Telengana, India; e Choithram Hospital and Research Centre, Indore, Madhya Pradesh, India; f Fortis Escorts Hospital, Jaipur, Rajasthan, India; g Gauhati Medical College, Guwahati, Assam, India; h Grant Medical College & JJ Hospital, Mumbai, Maharashtra, India; i Government Medical College & SAT Hospital, Thiruvananthapuram, Kerala, India; j IMS & SUM Medical College & Hospital, Bhubaneswar, Odisha, India; k Indira Gandhi Institute of Medical Sciences, Patna, Bihar, India; l Institute of Post Graduate Medical Education and Research, Kolkata, West Bengal, India; m King George’s Medical University, Lucknow, Uttar Pradesh, India; n Maulana Azad Medical College, Delhi, India; o Medanta– The Medicity, Gurgaon, Haryana, India; p MP Shah Government Medical College, Jamnagar, Gujarat, India; q PSG Institute of Medical Sciences, Coimbatore, Tamil Nadu, India; r Sher-i-Kashmir Institute of Medical Sciences, Srinagar, Jammu & Kashmir, India; s SCB Medical College and SVP Postgraduate Institute of Paediatrics, Cuttack, Odisha, India; t Agartala Government Medical College, Agartala, Tripura, India; u Ministry of Health & Family Welfare, Government of India, New Delhi, India; v World Health Organization, Geneva, Switzerland; w University of Basel Children’s Hospital, Basel, Switzerland; x Bill and Melinda Gates Foundation, India Country Office, New Delhi, India

**Keywords:** Intussusception, Retrospective surveillance, Children, Epidemiology, India

## Abstract

**Objective:**

Intussusception has been linked with rotavirus vaccine (RVV) as a rare adverse reaction. In view of limited background data on intussusception in India and in preparation for RVV introduction, a surveillance network was established to document the epidemiology of intussusception cases in Indian children.

**Methods:**

Intussusception in children 2–23 months were documented at 19 nationally representative sentinel hospitals through a retrospective surveillance for 69 months (July 2010 to March 2016). For each case clinical, hospital course, treatment and outcome data were collected.

**Results:**

Among the 1588 intussusception cases, 54.5% were from South India and 66.3% were boys. The median age was 8 months (IQR 6, 12) with 34.6% aged 2–6 months. Seasonal variation with higher cases were documented during March-June period. The most common symptoms and signs were vomiting (63.4%), bloody stool (49.1%), abdominal pain (46.9%) and excessive crying (42.8%). The classical triad (vomiting, abdominal pain, and blood in stools) was observed in 25.6% cases. 96.4% cases were diagnosed by ultrasound with ileocolic location as the commonest (85.3%). Management was done by reduction (50.8%) and surgery (41.1%) and only 1% of the patients’ died. 91.1% cases met Brighton criteria level 1 and 3.3% Level 2. Between 2010 and 2015, the case load and case ratio increased across all regions.

**Conclusion:**

Intussusception cases have occurred in children across all parts of the country, with low case fatality in the settings studied. The progressive rise cases could indicate an increasing awareness and availability of diagnostic facilities.

## 1 Introduction

Intussusception is the most common bowel obstruction in young children, usually occurring between 4 and 10 months of age [1], [2]. Some of these may be transient and resolve spontaneously. If the intussusception is not relieved early, it may lead to bowel ischemia and perforation and may be fatal [2]. Ileocecal region is the most common site documented. Some anatomical lead points may trigger in 10–15% cases [2]. Intussusception has been linked as an adverse reaction with rotavirus vaccine (RVV). An increased rate of intussusception (1 additional case per 10000 vaccinated children) following introduction of RVV, Rotashield^TM^ (Wyeth Lederle, Marietta, PA, USA) in 1999 forced its withdrawal from the market [3], [4], [5]. Recognising the intussusception risk, subsequent two candidate RVVs underwent clinical trials with over 60,000 participants documented no significant increased risk [6], [7]. The post-licensure surveillance with two RVVs (Rotarix^TM^, GlaxoSmithKline Biologicals, Rixensart, Belgium and Rotateq^TM^, Merck & Co. Inc., Kenilworth, USA) reported variable risks of intussusception across countries; ranging from no increased risk [USA [8] and Brazil [9] to a low risk of 1–2 additional cases per 100,000 vaccinated infants [Mexico [9] and Australia [10]. Introduction of RVV in national immunization programmes (NIPs) has been recommended by World Health Organization (WHO) to reduce the rotavirus diarrhoea related deaths. Also, WHO has recommended documentation of baseline rates of intussusception to allow for estimation of potential attributable risks associated with vaccine, to enable a quantitative assessment of the potential risks and benefits of a vaccine programme [11].

According to WHO, 96 countries have introduced RVV in their NIP till August 2018. India has introduced the RVV in NIP since April 2016 in a phased manner. Monitoring the risk of intussusception related to RVV requires reliable surveillance systems. Currently available studies from India have assessed the risk of intussusception from single or few medical centres. The studies use different case definitions and age groups, reference periods, methodology and case management protocols [12], [13], [14]. Measured intussusception incidence in children from published reports varied between 17.7 (95% CI 5.9, 41.4) to 254 (95%CI 5.9, 41.4) cases per 100,000 child-years [15], [16].

Wide variations in incidence and case load exist across sites in India and globally [17] although reasons for those variations are not known. There is limited information about regional differences in intussusception incidence among children from India. In view of the limited background data, we established a network of tertiary care hospitals to monitor trends of intussusception over time to identify any vaccine associated risk [18], [19]. In this study we report on the epidemiology of intussusception in under-two children prior to RVV introduction in India.

## 2 Methods

### 2.1 Study area and participating hospitals

This retrospective study of hospital-based sentinel surveillance for intussusception in under-two children was conducted at 19 Indian major tertiary care hospitals. Data were collected for a 69-months period (July 2010 to March 2016) at all these sites. The sites were selected from four regions (north, south, east, and west) of the country with three to six hospitals per region with a mix of medical colleges and private-sector hospitals (North region- 5 sites, 3 public and 2 private; South region- 5 sites, 2 public and 3 private; East region- 6 sites, 5 public and 1 private; West region- 3, public 2, and private 1). The data was collected during January 2016- September 2017. The method of selection of the sites are described in the protocol published [20].

### 2.2 Case definition, case selection and data collection

Children aged >1 month and <24 months (2–23 months) admitted to these hospitals with intussusception were reviewed. At the institutions using international classification of diseases (ICD), cases with one of the clinical conditions as per ICD-10 (K56.1, K56.2, K56.3, K56.4, K56.5, K56.6, K56.7 and K56.0) or ICD 9 (560.0, 560.2, 560.31, 560.30, 560.81, 560.9 and 560.1) were identified. At the institutes not using ICD classification, cases with exit diagnoses including intussusception, acute intestinal obstruction, subacute intestinal obstruction, acute abdomen, and blood in stool with vomiting were identified. Additionally, all the registers from clinical (pediatrics, pediatric surgery and emergency) wards, operation theatres, radiology (ultrasound, barium studies and CT scan) and pathology departments were screened to identify any cases. The case records of these suspected patients were extracted and reviewed to identify intussusception cases. For confirmed cases, data including demography, clinical features, management, and final outcome were abstracted using case record form (CRF). The data including symptoms, signs and other relevant information as documented in the case records were captured. A record of cases screened, identified as suspected and confirmed cases was maintained. For the suspected and confirmed cases with incomplete information from case records, the other sources of information were searched and supplemented. The modes of treatment cases as documented in case record were recorded (surgical- laparotomy with or without resection, reduction- hydrostatic or barium enema under ultrasound guidance or conservatively- managed for intestinal obstruction without reduction or surgery). The cases with documented findings compatible with intussusception on ultrasound, CT scan, barium enema or surgery were considered as confirmed cases. An independent case adjudication committee (CAC), including paediatrician, paediatric surgeon and radiologist, reviewed and assigned diagnostic certainty level using Brighton Collaboration criteria [1].

### 2.3 Quality assurance

To ascertain protocol adherence, rigor and completion of surveillance, multilevel quality assurance processes were implemented. Technical Advisory Group (TAG) members visited sites to assess the suspected cases identification, record retrieval and data abstraction for confirmed cases. Data team members visited all the sites on a second occasion, repeated the case identification process for 2014, 2015, and 2016 using the protocol to assess the completeness of data collection and identify any missed cases. The data abstracted for confirmed intussusception cases were verified from case records. Any additional data query was clarified with reference to the source documents.

### 2.4 Data management and analysis

Double data entry was done for the CRFs using a customised data entry platform with inbuilt data matching feature. The verified data were stored in a server with authorised access and daily backup. The descriptive analysis was included proportions for categorical; means and standard deviations for normally distributed continuous variables, and median with IQR for continuous variables with skewed distributions. The values between groups were compared for statistical significance using Student’s *t* test or Mann–Whitney *U* test depending on the skewness. Statistical significance was considered if p < 0.05. Statistical analysis was performed using STATA version 15.0 (Stata Corp LLC, Texas, USA). Due to the lack of well-defined population catchment area for the hospitals, the estimation of intussusception incidence was not feasible. Instead, we estimated intussusception case ratio per 1000 paediatric hospitalisations at each hospital for comparing the case volumes by sites and years. On review, it was observed that the paediatric surgery admission numbers were relatively stable compared to the medicine department and the intussusception cases were primarily admitted to the paediatric surgery wards. Thus the intussusception case ratio was estimated per 1000 paediatric surgery admissions at the hospitals for comparison and trend analysis. The detailed methodology can be referred from the published protocol [20].

### 2.5 Ethical issues

The study protocol was reviewed and approved by all the ethics review committees of each participating institute. Confidentiality in data handling was maintained.

## 3 Results

**Fig. 1:**
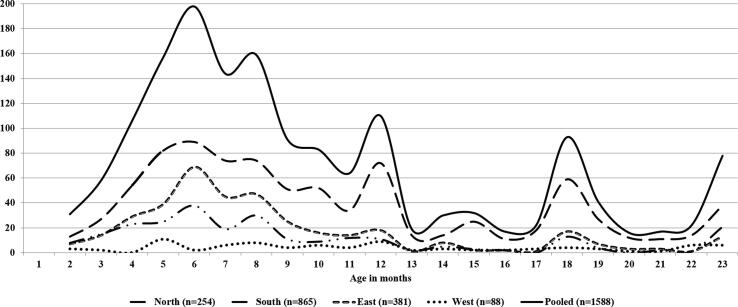
Age distribution of children aged 2–23 months with intussusception in India (region wise and pooled).

A total of 1588 children aged under two years (2–23 months) with intussusception were admitted to these hospitals between July 2010 and March 2016. Out of these 96 (6%) (North region, n = 13; South region, n = 68; East region, n = 10 and West region, n = 5) cases had past history of intussusception documented in the case records. Over half of the cases (n = 865, 54.5%) were documented from the hospitals in Southern region. Figures for other regions were: Eastern (n = 381, 24%), Northern (n = 254, 16%) and Western (n = 88, 5.5%). Demographic and clinical characteristics of the intussusception patients are summarised in Table 1. A consistent male predominance was observed across all regions (male: female ratios: pooled 1.96; north 3.0; south 1.65, east 2.2; and west 2.1). The majority of cases were aged <12 months, with 34.6% in the 2–6 months and 41% in the 7–12 months age bands. Nearly half (48.1%) of the patients were aged 4–8 months. Over one-third (34.7%) children were aged >1–6 months, the age for administration of routine RVV doses. The overall median age at presentation was 8 months (Inter Quartile Range, IQR 6, 12) with 8 months (IQR 6, 12) for boys and 8 months (IQR 6, 15) for girls (p = 0.09). The median age was higher for Western (11 months, IQR 7, 17) and Southern (9 months, IQR 6, 14) regions compared to Northern (7 months, IQR 5, 12) and Eastern (7 months, IQR 6, 10) regions (p = 0.00) (Table 1 and Fig. 1). Additional peaks in case load was observed around the 12th and 23rd months of age across all regions.

**Table 1:** Demographic and clinical characteristics of children aged 2-23 months with intussusception in India (region wise and pooled).

Variable	Category	North (n = 254) n (%)	South (n = 865) n (%)	East (n= 381) n (%)	West (n= 88) n (%)	Total (n= 1588) n (%)	P value
Age (in months)	2–6	109 (42.9)	265 (30.6)	158 (41.5)	18 (20.5)	550 (34.6)	0.00
	7–12	92 (36.2)	357 (41.3)	165 (43.3)	37 (42.1)	651 (41)	0.35
	13–18	25 (9.8)	141 (16.3)	31 (8.1)	16 (18.2)	213 (13.4)	0.00
	19–23	28 (11)	102 (11.8)	27 (7.1)	17 (19.3)	174 (11)	0.01
Gender	Male	191 (75.2)	539 (62.3)	263 (69)	60 (68.2)	1053 (66.3)	0.00
	Female	63 (24.8)	326 (37.7)	118 (31)	28 (31.8)	535 (33.7)	0.00
Place of residence	Same district	89 (35)	500 (57.8)	48 (12.6)	62 (70.5)	699 (44)	0.00
	Other districts@	127 (50)	342 (39.5)	331 (86.9)	24 (27.3)	824 (51.9)	0.00
	Outside state	38 (15)	23 (2.7)	2 (0.5)	2 (2.3)	65 (4.1)	0.00
Referral status	Primary*	188 (74)	625 (72.3)	334 (87.7)	72 (81.8)	1219 (76.8)	0.00
	Referred**	66 (26)	240 (27.8)	47 (12.3)	16 (18.2)	369 (23.2)	0.00
Symptoms	Vomiting¥	164 (64.6)	631 (73)	145 (38.1)	66 (75)	1006 (63.4)	0.00
	Bilious vomiting¥¥	28 (11)	100 (11.6)	24 (6.3)	14 (15.9)	166 (10.5)	0.01
	Abdominal pain	144 (56.7)	412 (47.6)	136 (35.7)	53 (60.2)	745 (46.9)	0.00
	Excessive crying	42 (16.5)	538 (62.2)	70 (18.4)	30 (34.1)	680 (42.8)	0.00
	Abdominal distension	95 (37.4)	60 (6.9)	85 (22.3)	15 (17.1)	255 (16.1)	0.00
	Rectal bleeding	135 (53.2)	456 (52.7)	141 (37)	48 (54.6)	780 (49.1)	0.00
	Diarrhoea	33 (13)	177 (20.5)	53 (13.9)	30 (34.1)	293 (18.5)	0.00
	Constipation	47 (18.5)	84 (9.7)	25 (6.6)	12 (13.6)	168 (10.6)	0.00
	Fever	49 (19.3)	151 (17.5)	41 (10.8)	19 (21.6)	260 (16.4)	0.01
	Lethargy	9 (3.5)	50 (5.8)	14 (3.7)	8 (9.1)	81 (5.1)	0.09
	Classical triad#	64 (25.2)	255 (29.5)	65 (17.1)	23 (26.1)	407 (25.6)	0.00
Signs	Pallor	29 (11.4)	22 (2.5)	34 (8.9)	11 (12.5)	96 (6.1)	0.00
	Dehydration	27 (10.6)	66 (7.6)	44 (11.6)	8 (9.1)	145 (9.1)	0.13
	Fever	16 (6.3)	92 (10.6)	36 (9.5)	9 (10.2)	153 (9.6)	0.23
	Lethargy	22 (8.7)	53 (6.1)	21 (5.5)	4 (4.6)	100 (6.3)	0.38*
	Abdominal distension	96 (37.8)	74 (8.6)	84 (22.1)	20 (22.7)	274 (17.3)	0.00
	Abdominal tenderness	46 (18.1)	69 (8)	35 (9.2)	12 (13.6)	162 (10.2)	0.00
	Abdominal mass	41 (16.1)	376 (43.5)	24 (6.3)	15 (17.1)	456 (28.7)	0.00
	Bowel sound (absent/ abnormal)	35 (13.8)	17 (2)	18 (4.7)	6 (6.8)	76 (4.8)	0.00
	Rectal prolapse	51 (20.1)	0 (0.0)	4 (1.1)	0 (0.0)	55 (3.5)	0.00*
	Rectal mass	23 (9.1)	8 (0.9)	6 (1.6)	1 (1.1)	38 (2.4)	0.00*
	Blood on rectal examination	80 (31.5)	107 (12.4)	75 (19.7)	31 (35.2)	293 (18.5)	0.00
	Triad (sign/symptom) ##	147 (57.9)	464 (53.6)	148 (38.9)	48 (54.6)	807 (50.8)	0.00
Diagnosis	Ultrasound	221 (87)	862 (99.7)	364 (99.5)	84 (99.5)	1531 (96.4)	0.00*
	CT scan	1 (0.4)	0 (0.0)	0 (0.0)	1 (1.1)	2 (0.1)	0.02*
	Barium enema	0 (0.0)	0 (0.0)	2 (0.5)	1 (1.1)	3 (0.2)	0.03*
	Surgery	32 (12.6)	3 (0.4)	15 (3.9)	2 (2.3)	52 (3.3)	0.00*
Intussusception location	Colo-colic	7 (2.8)	22 (2.5)	5 (1.3)	10 (11.4)	44 (2.8)	0.00
	Ileo-colo-colic	18 (7.1)	11 (1.3)	6 (1.6)	3 (3.4)	38 (2.4)	0.00*
	Ileo-ileal	40 (15.8)	18 (2.1)	7 (1.8)	10 (11.4)	75 (4.7)	0.00
	Ileo-ileo-colic	9 (3.5)	27 (3.1)	9 (2.4)	2 (2.3)	47 (3)	0.83*
	Ileo-colic	173 (68.1)	775 (89.6)	348 (91.3)	58 (65.9)	1354 (85.3)	0.00
	Jejuno-jejunum	1 (0.4)	4 (0.5)	0(0.0)	2 (2.3)	7 (0.4)	0.06*
	>1 location€	6 (2.4)	8 (0.9)	6 (1.6)	3 (3.4)	23 (1.5)	0.10*
Pathological lead point (PLP)	Lymph node	66 (26)	74 (8.6)	15 (3.9)	33 (37.5)	188 (11.8)	0.00*
	Appendix	8 (3.1)	5 (0.6)	39 (10.2)	2 (2.3)	54 (3.4)	0.00*
	Payer's patch	2 (0.8)	10 (1.2)	2 (0.5)	0 (0)	14 (0.9)	0.06*
	Polyp	0 (0)	2 (0.2)	1 (0.3)	0 (0)	3 (0.2)	0.65*
	Others&	9 (3.6)	12 (1.3)	3 (0.8)	1 (1.1)	25 (1.5)	0.17
	Any lead point	85 (33.5)	103 (11.9)	60 (15.8)	36 (40.9)	284 (17.9)	0.00
Treatment	Reduction	47 (18.5)	686 (79.3)	34 (8.9)	39 (44.3)	806 (50.8)	0.00
	Surgery	187 (73.6)	165 (19.1)	273 (71.7)	28 (31.8)	653 (41.1)	0.00
	Conservative	19 (7.5)	14 (1.6)	61 (16)	20 (22.7)	114 (7.2)	0.00
	None$	1 (0.4)	0 (0.0)	13 (3.4)	1 (1.1)	15 (0.9)	0.00*
Surgery type£	No resection	77 (30.3)	118 (13.6)	202 (53)	21 (23.9)	418 (26.3)	0.00
	With resection	110 (43.3)	47 (5.4)	71 (18.6)	7 (8)	235 (14.8)	0.00
Outcome	Discharged	248 (97.6)	863 (99.8)	341 (89.5)	87 (98.9)	1539 (96.9)	0.00*
	Referred	0 (0)	0 (0)	0 (0)	0 (0)	0 (0)	-
	LAMA^	3 (1.2)	0 (0.0)	29 (7.6)	1 (1.1)	33 (2.1)	0.00*
	Died	3 (1.2)	2 (0.2)	11 (2.9)	0 (0.0)	16 (1)	0.00*
Brighton Criterial level	Level 1	234 (92.1)	851 (98.4)	307 (80.6)	67 (76.1)	1459 (91.9)	0.00
	Level 2	10 (3.9)	9 (1)	23 (6)	11 (12.5)	53 (3.3)	0.00
	Level 3	0 (0.0)	0 (0.0)	0 (0.0)	0 (0.0)	0 (0.0)	-
	Not fitting any level	10 (3.9)	5 (0.6)	51 (13.4)	10 (11.4)	76 (4.8)	0.00

Notes:

PR: Per-rectal

The p values for some parameters could not be estimated due to no value in at least two groups.

* The patient presented to the study hospital as the first institute.

** The patient referred to the study hospital from another hospital.

¥ Vomiting includes bilious vomiting.

¥¥ Includes only bilious vomiting.

# Classical triad include abdominal pain, vomiting and blood in stools

## Triad include abdominal pain, vomiting and rectal bleeding (detected either as blood in stool or blood on per-rectal examination)

$ None, as the patient(s) were referred or died before any definite treatment.

£ The denominators used for percentage estimation are patients undergone surgery only.

^ LAMA: Left against medical advice.

@ The residence of the patient is in other district in the state where the study hospital is based.

€ The location of intussusception is at more than one site; such as Ileo-colic + Colo-colic, Ileo-olic + Ileo-colo-colic, Ileo-colic + Ileo-ileo-colic, Ileo-colic+ Ileo-ileal.

& Others including cyst, Meckel's diverticulum, incisional hernia, intraluminal growth and malrotation.

**Fig. 2:**
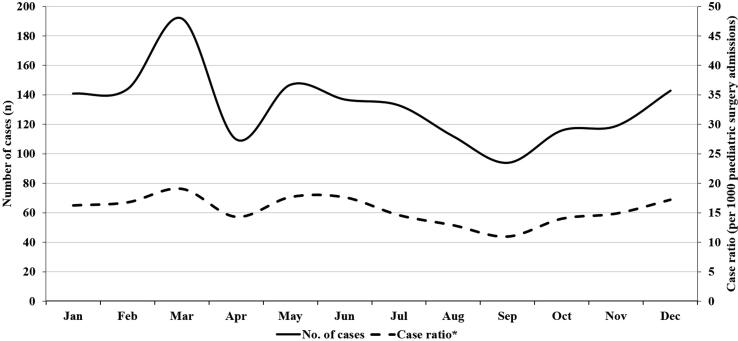
Calendar monthly distribution of intussusception case load and case ratio in children aged 2–23 months in India (pooled).

Seasonal variation was observed with higher number of intussusception cases and case ratio during March to June months over each individual year studied (Fig. 2).

**Fig. 3:**
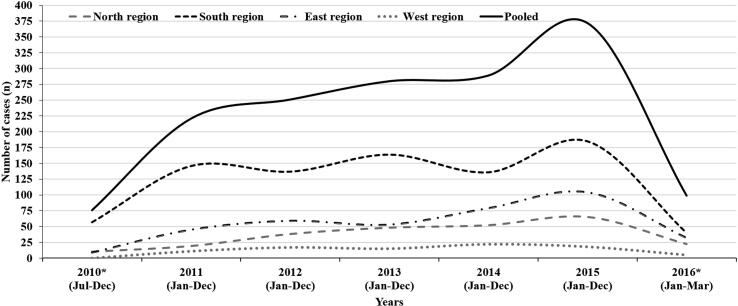
Trend of intussusception case load in children aged 2–23 months in India during 2010–2016 (region wise and pooled).

**Fig. 4:**
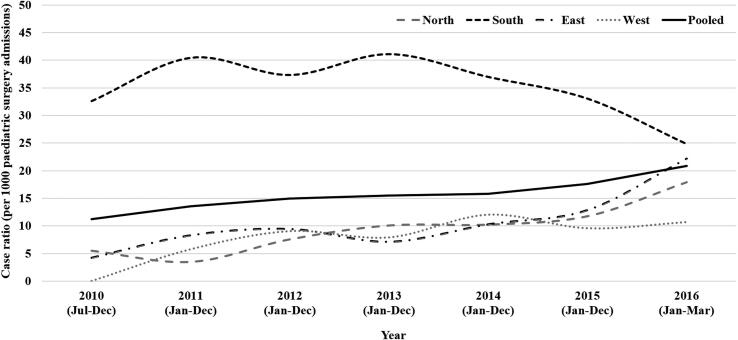
Trend of intussusception case ratio in children aged 2–23 months in India during 2010–2016 (region wise and pooled).

While the pooled intussusception case ratio was 15.5 (IQR 14.8, 16.3) per 1000 paediatric surgery admissions, the case ratio for Northern, Southern, Eastern and Western regions were observed to be 8.8 (IQR 7.8, 9.9), 36.2 (IQR 33.9, 38.6), 9.9 (IQR 8.9, 10.9) and 8 (IQR 6.5, 9.9) respectively. Between 2010 and 2015, rises in both absolute number of intussusception cases and case ratio (per 1000 paediatric surgery admissions) was observed across all the regions, but the rise was relatively higher for Eastern and Northern than the Southern and Western regions (see Fig. 3, Fig. 4).

While 51.9% of hospitalized patients resided outside the hospital district, 44% were from the same district and 4.1% came from other states. Overall 76.8% cases presented directly to hospitals and 23.2% patients were referred from other hospitals. The median duration of symptoms to admission was 3 days (IQR 1, 5 days), but a shorter interval was observed in Southern (median 1 day, IQR 1, 5 days) and Western (median 2 days, IQR 1, 6 days) than in Northern and Eastern (median 3 days, IQR 1, 10 days) (p = 0.04) regions.

The most common symptoms were vomiting (63.4%), passage of blood stained stool (49.1%), abdominal pain (46.9%) and excessive crying (42.8%). Bilious vomiting was documented in 10.5% cases, more frequently Western region (15.9%). Higher proportions of abdominal pain in Western (60.2%) and Northern (56.7%); excessive crying in Southern (62.2%); blood in stool in Northern, Southern and Western (52.7%-54.9%) regions were documented. The intussusception classical symptoms triad (abdominal pain, vomiting and blood in stools) was documented in 25.6% cases, which varies from 25.2% to 29.5% in in Northern, Southern and Eastern regions, but was quite lower (17.1%) in the Western region. There was no variation in the presence of the symptoms triad with the interval between onset of symptoms and presentation to the hospital. On examination children had abdominal mass (28.7%), abdominal distention (17.3%), blood on per-rectal examination (18.5%), and abdominal tenderness (10.2%). The prevalence of findings varied across regions for abdominal distention (Northern 37.8% to Southern 8.6%), abdominal mass (Southern 43.5% to Eastern 6.3%), and blood on per-rectal examination (Western 35.2% to Northern 12.4%). On considering presence of blood on per-rectal examination, presence of triad of intussusception (abdominal pain, vomiting and blood in stools or per-rectal examination) was present in 50.8% and ranged from 57.9% in Northern to 38.9% in Eastern regions.

Most cases were diagnosed by ultrasound (96.4%) and few by laparotomy (3.3%). The most common site of intussusception was ileocolic (85.3%) across all regions, age groups and gender. Other intussusception sites included ileo-ileal (north 15.8% and west 11.4%), colo-colic (west 11.4%) and ileo-colo-colic (north 7.1%). Double site intussusception was documented in few (1.5%) cases. Pathological lead point (PLP) was documented in 91 (17.9%) cases with lymph nodes (11.8%) as the most common one and higher proportion in Western region (37.5%) followed by Northern region (26%).

Most patients were treated by reduction (50.8%) and surgery (41.1%), while 7.2% children were managed conservatively. Among children managed by surgery, 14.8% required bowel resection. The median interval to presentation was not associated with different treatment choices: surgery (1 day; IQR 0, 3), reduction (1 day; IQR 1, 2) and conservative treatment (1 day; IQR 0, 3). Higher proportion of patients underwent surgery in Northern (73.6%) and Eastern (71.7%) regions compared to Western (31.8%) and Southern (19.1%) regions. Five hospitals did not use reduction (3 in Eastern, 1 in Northern and 1 in Southern regions) but, instead, use exclusive surgery. At the hospitals with both surgical and reduction facilities for management, surgery was done in 28.3% (341/1205) cases with 18.1% cases reduced under anaesthesia and the remainder required bowel resection. The indications for surgery were failed reduction (33.9%), late presentation (23%) and associated complications (43.1%). Only 1% of the cases died and most of these presented late (median interval 3 days, IQR 1, 6 days). The causes of death were due to complications (sepsis, shock, and gangrene) associated with delayed presentation and after surgery. Thirty three cases left against medical advice before definite treatment. The overall median period of hospitalisation was 3 days (IQR 2, 6 days), which varied from 2 to 6 days; there were important regional variations, from 2 days in the Southern to 6 days in the Northern and Eastern regions. The median hospitalisation periods were 7 days (IQR 5, 9 days), 2 days (IQR 2, 3 days) and 2 days (IQR 2, 4 days) for surgery, reduction and conservative management respectively. Using Brighton diagnostic criteria, 91.1% cases were categorised as Level 1, 3.3% as Level 2 and none in Level 3. No level could be assigned for 4.8% cases.

## 4 Discussion

In preparation for RVV introduction, assessing the background rate of intussusception helps prepare monitoring vaccine safety [21], [22]. The global average rate of intussusception is about 74 (range 9–328) per 100,000 child years based on review of 82 studies [17]. Very high incidences have been measured in South Korea [23], Vietnam [24], Israel [25], and Japan [26] with 328, 302, 219, and 185 per 100,000 child years respectively [17]. Reported intussusception rates in India vary from 17.7 to 254 per 100,000 child years, [15], [16]. Most reports from India are hospital-based passive surveillance and few are population-based active surveillance. These studies were of limited geographical representation to derive any national interpretation. The RVV pre-licensure studies in India (for Rotavac^TM^ and Rotasiil^TM^) have not shown increased intussusception risk.

This study was undertaken to document the intussusception case load in India through a nationally representative sentinel surveillance network of tertiary care hospitals to assist monitoring the trend following RVV introduction. We observed important regional difference in intussusception case load and case ratio. The Southern region documented higher absolute case number (3–10 times) and case ratio (4–5 times). The higher number of cases and case ratio in southern region sites could be due to better access to diagnostic facilities and timely referral. Additionally presence of pediatric surgeon and management facility could be the other reasons for higher referral to some of these hospitals. Higher case load and case ratio were observed during March to June, as previously reported from India [14], [16], [27], [28].

Actual case incidence could not be estimated due to lack of defined draining population base. Instead, we used the intussusception case ratio per 1000 paediatric surgery admissions which were comparable to absolute case load. This proxy could be useful for comparison across the sites or regions and tracking in future studies. Three-fourth of the diagnosed cases were during the first year of life, with a majority of male patients aged 4–8 months, which was similar to other reports from India [12], [14], [16], [27] and globally [1], [12], [14], [16], [17], [27], [29], [30]. The variations in the symptoms and signs may be due to the interval between onset and admission time, longer in north and east regions. In 25.6% cases, the intussusception triad (abdominal pain, vomiting and blood in stools) was documented, which was higher than reports from other parts of India [14], [27]. When either symptom (blood in stool) or sign (blood on per-rectum examination) was considered, the prevalence of a triad was 50.8%. There was regional variation in the observation of a triad which also varied with the interval prior to presentation. The most common site for intussusception was ileocolic, which matched with reports from India [12], [16], [27] and globally [17]. PLPs were documented in 17.9% patients and lymph nodes were the most common (11.8%). Cases that presented early had shorter hospital stay. While in several hospitals reduction was the leading treatment modality, surgery was the only treatment method at some of the hospitals. A high degree of suspicion and early investigation for detection with appropriate referral is crucial for reducing surgical intervention. The proportion of cases with level 1 certainty according to BC criteria was high and similar to other reports from India [5], [16], [27]. For some cases Level 1 certainty could not be achieved due to non-documentation of reduction. Based on the findings of this study, the available information with the risk of intussusception with the RVVs in market and age pattern from literature, the pattern (age of occurrence, seasonality and sites) should remain similar. The studies after RVV introduction in India shall inform on this.

The study has several limitations. These sentinel surveillance sites were tertiary care hospitals and had no definite draining population territory. Thus estimation of incidence or a case rate was not possible. Several of the institutions used manual and paper record keeping methods and were not practicing the ICD classification system. This study being retrospective in nature, could have missed the cases, if they were not listed or available in the record section. The data collection may have missed the data including symptoms, signs and other information, if not documented in the case records. The number of institutes across the regions, the case load at the hospitals, the methods of case record archival and listing of the diagnoses might have influenced the case yield.

This study documented the intussusception case load, their distribution, clinical features, management and outcome across different sites and regions in India before RVV introduction. This may provide a reasonable background information. More importantly it establishes the methodological basis for active surveillance of intussusception following RVV introduction. The private and public mix of hospitals and regional representation will allow for a more representative evaluation of the vaccines in the whole population. Intussusception is a common condition among children under-two years of age across in India with majority of cases during infancy. There were regional variations in the case load and ratio observed. An increasing secular trend of intussusceptions has been documented among children across all regions prior to the beginning of the rotavirus vaccination program. Case management was dependent on facilities available at the hospitals. Although mortality was low, it can be further prevented by early case detection, referral and timely management. Studies of intussusception following vaccine introduction will require RVV exposure documentation. A periodic monitoring of the intussusception should be undertaken to interpret the trend over time. It may also be useful to further investigate the potential risk factors for the regional variations and increasing trend.

## Funding

This project was funded by the 10.13039/100000865Bill and Melinda Gates Foundation, 10.13039/100011408USA (grant number OPP1116433).

## Ethics approval

The study protocol was reviewed and approved by INCLEN Ethics Committee (Ref: IIEC 023) and all participating Study Site Institute Ethics Committees.

## Data availability

The data can be made available in request from the corresponding author.

## Authors’ contributions

MKD and NKA conceptualised the framework for the study protocol, training, data analysis and interpretation. All TAG members provided input for finalisation of the study protocol and provided quality assurance oversight. All study site investigators led and supervised the data collection. BG and AS coordinated the data collection and collation. MKD and BG analysed the data. MKD and BG wrote the first draft of the manuscript. All authors reviewed, provided critical input and approved the final version. The content represents the views of the authors alone and do not necessarily represent the official positions of their organizations, World Health Organization or Ministry of Health and Family Welfare, Government of India.

## Declaration of Competing Interest

The authors declare that they have no known competing financial interests or personal relationships that could have appeared to influence the work reported in this paper.
