# Supplementary figures and images for: Intussusception in children aged under two years in India: Retrospective surveillance at nineteen tertiary care hospitals
Source: Vaccine. 2020 Oct 7;38(43):6849–57. doi: 10.1016/j.vaccine.2020.04.059 (PMC7528221; doi:10.1016/j.vaccine.2020.04.059)

**SUPPLEMENTARY FIGURES**

Supplementary Figure S1: The flow of case screening and recruitment


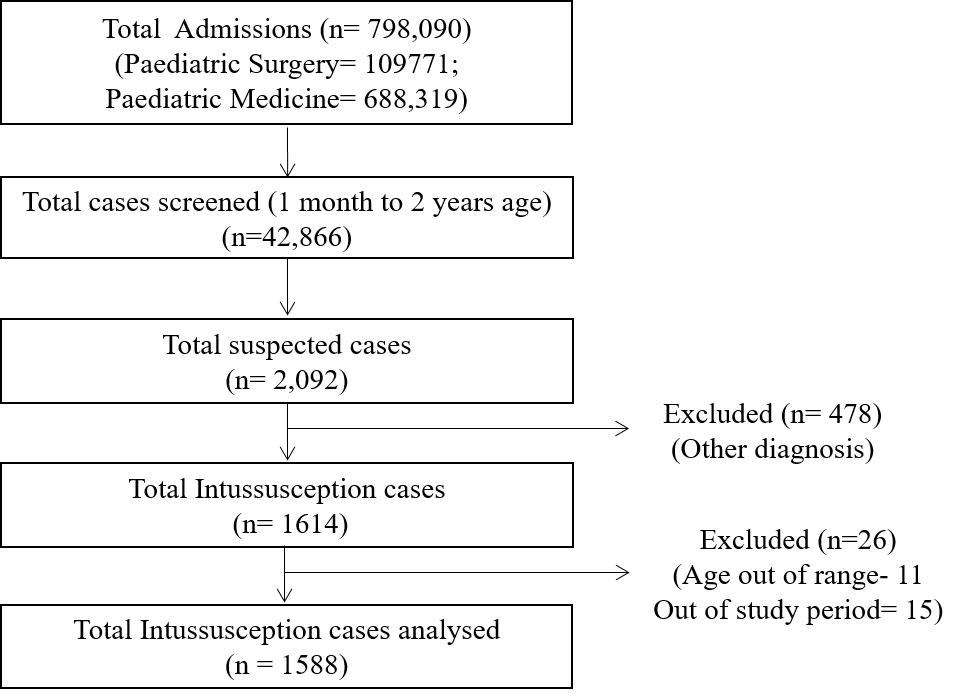

Supplement: Supplementary data 2 [file mmc2.docx]
